# Tracing the Origin and Spread of Agriculture in Europe

**DOI:** 10.1371/journal.pbio.0030410

**Published:** 2005-11-29

**Authors:** Ron Pinhasi, Joaquim Fort, Albert J Ammerman

**Affiliations:** **1**School of Human and Life Sciences, Whitelands College, Roehampton University, London, United Kingdom,; **2**Departament de Fisica, E.P.S. P-II, Universitat de Girona, Campus de Montilivi, Catalonia, Spain,; **3**Department of Classics, Colgate University, Hamilton, New York, United States of America; The Wellcome Trust Sanger InstituteUnited Kingdom

## Abstract

The origins of early farming and its spread to Europe have been the subject of major interest for some time. The main controversy today is over the nature of the Neolithic transition in Europe: the extent to which the spread was, for the most part, indigenous and animated by imitation (cultural diffusion) or else was driven by an influx of dispersing populations (demic diffusion). We analyze the spatiotemporal dynamics of the transition using radiocarbon dates from 735 early Neolithic sites in Europe, the Near East, and Anatolia. We compute great-circle and shortest-path distances from each site to 35 possible agricultural centers of origin—ten are based on early sites in the Middle East and 25 are hypothetical locations set at 5° latitude/longitude intervals. We perform a linear fit of distance versus age (and vice versa) for each center. For certain centers, high correlation coefficients (*R* > 0.8) are obtained. This implies that a steady rate or speed is a good overall approximation for this historical development. The average rate of the Neolithic spread over Europe is 0.6–1.3 km/y (95% confidence interval). This is consistent with the prediction of demic diffusion (0.6–1.1 km/y). An interpolative map of correlation coefficients, obtained by using shortest-path distances, shows that the origins of agriculture were most likely to have occurred in the northern Levantine/Mesopotamian area.

## Abstract 2

An analysis of radiocarbon dates from early Neolithic sites reveals that agriculture in Europe most likely originated in the northern Levant and Mesopotamia and spread by population growth and migration, rather than by cultural diffusion.

## Introduction

The study of the origins of farming in the Near East and its dispersal to Europe has been a subject of major interest to archaeologists, anthropologists, linguists, and geneticists. The interest in agricultural origins can be traced to Gordon Childe [1], who proposed in 1942 that the Neolithic populations of the Near East were under substantial economic and demographic pressures triggered by marked population growth following the successful development of the Neolithic lifestyle. In his later book *The Dawn of European Civilisation* [2], Childe applied his demographic/Malthusian concept of population pressure and territorial expansion to the study of Neolithic Europe, asserting that the first Neolithic crops and domesticated animals did not reach Europe by means of trade or exchange but by means of migration or the colonization of farmers and shepherds from the Near East.

Clark [3,4] was the first to study the Neolithic dispersal by looking at the spatiotemporal pattern of radiocarbon dates in Europe and the Near East. Clark allocated the few carbon-14 dates available at the time to three temporal classes: Group 1, dates equal to or earlier than 5,200 BC; Group 2, dates between 5,200 and 4,000 BC; and Group 3, dates between 4,000 and 2,800 BC. His map shows a basic trend from east to west for the early Neolithic in Europe that is consistent with Childe's ideas [2].

In 1971, the first quantitative analysis of the spread of early farming in Europe was undertaken by Ammerman and Cavalli-Sforza [5], who then went on to develop a new perspective on the processes at work behind the Neolithic dispersal [6]. To measure the average rate of spread, Ammerman and Cavalli-Sforza [5] collected the radiocarbon dates from 53 early Neolithic sites, which were representative of the arrival of early farming in different parts of Europe, and performed a regression analysis. Four archaeological sites in the Near East (Jericho, Jarmo, Çayönü, and Ali Kosh) were taken as probable centers of agriculture—a fifth center used in their study was the center of gravity of their four sites—and the great-circle distance from each European site to a given center was then calculated. Diffusion rates for the respective centers were obtained from linear fits of the radiocarbon ages and the geographic distances. The results from all of the centers gave an average rate of about 1 km/y. The correlation coefficients were relatively high (*R* > 0.8) for each of their five centers, indicating that a regular rate offers a good overall description of the spread.

Ammerman and Cavalli-Sforza [6,7] stressed that, in principle, the observed rate could be explained as the consequence of cultural diffusion (the spread of crops and farming technology without the movement of people) or demic diffusion (the spread of farmers themselves)—or even some combination of the two. At the same time, they were the first to emphasize the role of demic diffusion in the Neolithic transition and to draw attention to the agreement between the observed average rate of spread and the one predicted by a demic wave-of-advance model [7]. Their wave-of-advance model, borrowed from the field of population biology, proposes that active population growth at the periphery of the farmers' range, in combination with local migratory activity (isotropic in character), would produce a population range expansion that moves outwards in all directions and advances at a relatively steady rate. They also predicted that the mixing of Neolithic and Mesolithic populations would lead to genetic gradients with extreme gene frequencies in the areas with the oldest Neolithic sites [6]. This prediction was confirmed in 1978 by the analysis of classical genetic markers [8]. The first principal component of the classical polymorphisms shows a geographic cline, from the south-east to the north-west of Europe [9,10], as expected under the hypothesis of demic diffusion and the interaction of Neolithic and Mesolithic populations. However, it must be added that clines can arise through several processes [11,12]. Moreover, even if a genetic cline is associated with a demic-diffusion process, it does not in itself indicate the time in which it was established [13]. On the other hand, a strong correlation is observed between genetic and archaeological distances, and this correlation supports the hypothesis of demic diffusion [14].

Molecular studies using mitochondrial DNA, Y-chromosome DNA, and nuclear DNA differ in their assessment of the contribution of Near Eastern farmers to the European gene pool. Some mitochondrial-DNA studies suggest that the contribution of Near Eastern farmers to the European gene pool is about 20% [15,16]. A similar percentage (22%) is suggested by a Y-chromosome study carried out by Semino et al. [17]. However, the data in [17] were reexamined by Chikhi et al. [18], who found (through a different methodology) an average contribution of between 50% and 65% by Near Eastern farmers to the European gene pool. Estimations depend not only on the markers employed but also on the model used (and its inherent assumptions). A recent study that makes use of mitochondrial-DNA, Y-chromosome DNA, and other autosomal markers [19] finds that the Neolithic contribution is much higher than 20%, and decreases from east to west, as expected under the Near Eastern demic-diffusion model. Finally, nuclear-DNA studies support a substantial contribution of Near Eastern populations to the European gene pool [20]. Thus, many genetic studies tend to support the idea of demic diffusion at some level, but there is still a lack of consensus with regard to the percentage of the contribution of early Near Eastern farmers to the European gene pool (see also [21,22]).

Recent archaeobotanical, archaeological, and craniometric studies suggest that, in all probability, the spread of farming to Europe was a complex process, and these studies point to the occurrence of an “aceramic” or “pre-pottery” dispersal to Cyprus, Crete, and the Argolid from various locations in the Near Eastern zone [23–26]. These studies and others highlight the complexity of the historical process of the spread of farming, suggesting significant regional variations in the dispersal process, with varying degrees of demic diffusion and cultural diffusion.

Some points that have been relatively neglected are: (1) the identification of the area in the Near East from which the spread began; (2) the computation of a statistically-significant error range for the observed speed; (3) the computation of the speed range predicted by demic diffusion; (4) the comparison of these observed and predicted ranges; (5) the effect of the Mediterranean Sea as a barrier (by computing shortest-path in addition to great-circle distances); and (6) the calibration of dates. Below we address these issues.

Our work involves a reassessment of the wave-of-advance model using a sample of 735 dates from early Neolithic sites in Anatolia, the Near East, and Europe (Table S1 and Text S1). We try to take into account all available sites that have standard errors of the mean of less than 200 radiocarbon years, including those from the Alpine region and from various regions in the Near East. We assess the correlation coefficient, *R,* and the rate-of-advance parameters for 25 hypothetical centers of origin of agriculture (HOA) and ten probable centers of origin of agriculture (POA). The 25 HOA are defined solely on the basis of their geographic location. The ten POAs consist of nine archaeological sites that have yielded some of the earliest evidence for cereal domestication in the Near East, plus the center of gravity from the original analysis mentioned above [5]. We then calculate four sets of *R*-values (one for each distance and dating method) and apply statistical methods to these sets in order to determine: (1) the most likely average speed of the spread over Europe; (2) its error range; and (3) the most likely area of the origins of agriculture. We also compute the speed range predicted by a demic diffusion model and compare it to the range inferred from the observed data.

## Results

### Determination of the Observed Rate of Spread of Agriculture

**Figure 1:**
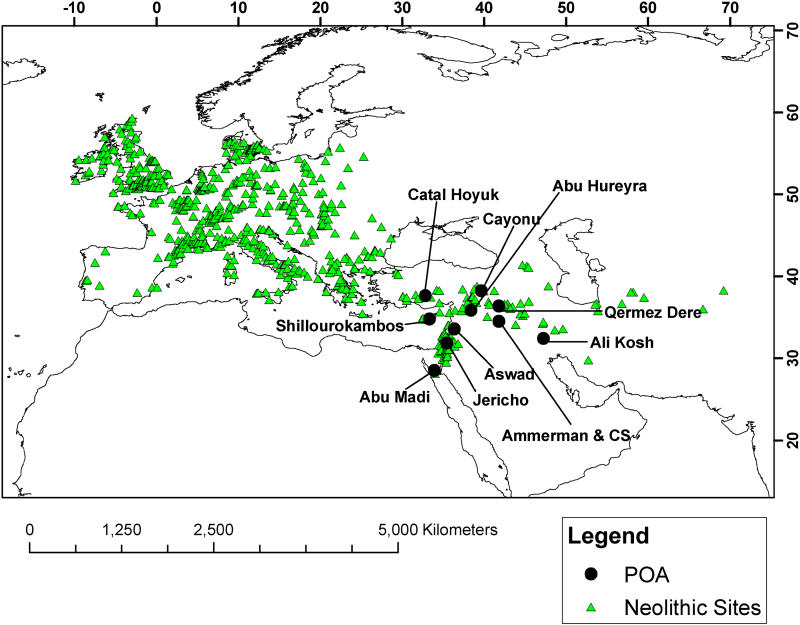
Location of the 735 Archaeological Sites Used in the Analysis as well as the Ten POAs Listed in Table 1

The values of the correlation coefficient, *R,* derived from the linear regressions for the ten POAs (Figure 1) are presented in Table 1. Let us first consider great-circle distances. The POA with the highest correlation coefficient is Center 3 (Abu Madi). However, eight out of the nine other POAs have values of *R* that overlap with the range for Abu Madi (*R* = 0.827 ± 0.026, using uncalibrated dates). Therefore, their *R*-values are similar, and they can be regarded as likely places of origin for the dispersal. Center 1 (Çatal Höyük) is the only POA with an *R*-range that does not overlap with that of the center with the highest value of *R*; it has a substantially lower value (using either uncalibrated or calibrated ages). Interestingly, Table 1 shows that this conclusion changes when we consider shortest-path distances (see Text S2 for details on the computation of shortest-path distances). When shortest-path distances are used, the center with the highest value of *R* is no longer the most southern one (Abu Madi) of the POAs shown in Figure 1. At the same time, all of the ten POAs now have overlapping ranges of *R*-values (using either uncalibrated or calibrated dates). In short, the analyses based on shortest-paths yield an area for the origins of agriculture located to the north of the one identified by the use of great-circle distances (this topic is analyzed in detail below).

**Table 1:**
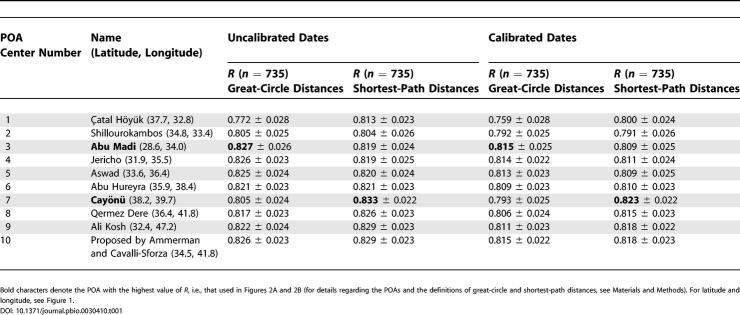
Several Sites That Have Been Considered as POAs to Europe, Their Locations, and Values of the Correlation Coefficient, *R,* Using the 735 Sites in Figure 1 (with 95% Confidence-Interval Errors, Obtained by Bootstrap Resampling)

**Figure 2:**
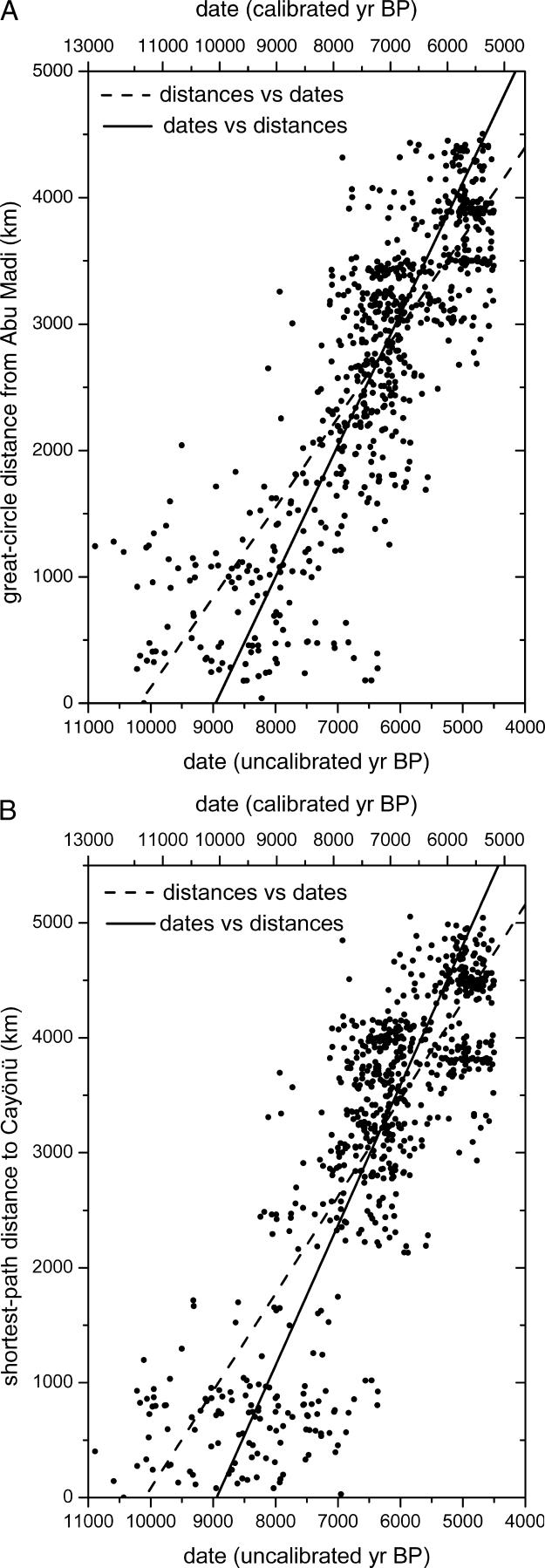
Linear Regression Fits to the Data (*n* = 735 Sites) for Uncalibrated Dates in Years BP and Distances of Sites Computed from the POA with the Highest *R*-Value in Table 1 (A) Based on great-circle distances. The speed implied by the distance-versus-time regression is the slope of the dashed line, namely 0.71 ± 0.04 km/y (in agreement with statistical theory, the error range of 0.04 km/y has been computed as twice the standard error of the slope and corresponds to a 95% confidence interval). The speed implied by the time-versus-distance regression (full line) is the inverse of the corresponding regression slope, namely 1.04 ± 0.05 km/y (95% confidence interval). Therefore, we estimate the overall speed range as 0.7–1.1 km/y. If calibrated dates are used in the analysis (top axis), the result is 0.6–1.0 km/y (see the first figure in Protocol S1). (B) Based on shortest-path distances. The distance-versus-time regression yields 0.85 ± 0.04 km/y, whereas the time-versus-distance regression yields 1.22 ± 0.06 km/y. The overall estimated speed range is thus 0.8–1.3 km/y. If calibrated dates are used (top axis), the result is 0.7–1.1 km/y (see the second figure in Protocol S1).

In order to estimate the speed of the agricultural wave of advance, we use distances relative to the POA with the highest *R*-values in Table 1: Abu Madi for great circles (Figure 2A) and Cayönü for shortest paths (Figure 2B). This yields a speed range of 0.7–1.1 km/y using great circles and 0.8–1.3 km/y using shortest paths (95% confidence interval, see the caption of Figure 2). The shortest-path rate is obviously higher because the corresponding distances are equal or longer than great-circle distances, but what is very interesting is that the speed range is almost identical whether we use great circles or shortest paths. This also holds if we use calibrated dates (which yield 0.6–1.0 km/y for great circles and 0.7–1.1 km/y for shortest paths; see the caption of Figure 2). All of the other POAs (Table 1) yield essentially the same speed range (0.6–1.3 km/y). The time at which the spread began can be estimated, under the same hypothesis of linearity (straight fits in Figure 2), to fall within the interval of 9,000–10,500 years before present (BP; uncalibrated years) or 10,000–11,500 BP (calibrated years).

### Speed Predicted by a Demic-Diffusion Model

The results from Figure 2 strongly suggest that the average rate of the Neolithic transition was in the range of 0.6–1.3 km/y, and that the advance of the front took a form that was approximately linear (*R* > 0.8). The spread of early agro-pastoralism swept over Europe, taken as a whole, essentially at a regular speed—a rate that shows no overall trend either toward acceleration or toward deceleration over time [7]. As explained below, this range of values is compatible with that predicted by the time-delayed theory of the Neolithic transition [27]. The time-delayed theory is nothing but a refinement of the wave-of-advance model developed by Ammerman and Cavalli-Sforza [6,7]. The refinement takes into consideration the diffusive delay which is due to the generation time during which children remain with their parents and do not relocate their place of residence [27]. The time-delayed theory agrees with other human and non-human range expansions [28–30], as well as with the spread of viral infections [31].

As far as we know, no cultural-diffusion model to date has been able to derive a speed compatible with the observed range (0.6–1.3 km/y). This is an important point that has been neglected in the literature up to now. In contrast, the time-delayed demic model [27] predicts that the speed is



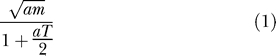



where *a* is the initial growth rate of the population number, *m* is the mobility, and *T* is the mean generation time [27]. The values of *a* and *m* have been carefully derived in previous work from plots of the population number versus time *(a)* and records of individual movements *(m)*. Data from anthropological studies gathered hitherto yield estimates of 0.029–0.035/y for *a,* 900–2,200 km^2^/generation for *m,* and 29–35 y for *T* (Text S3). Using these ranges, the above formula yields a speed range of 0.6–1.1 km/y. Thus, the speed range predicted by demic diffusion, namely 0.6–1.1 km/y, is compatible with that observed, namely 0.6–1.3 km/y (obtained above from Figure 2). Our conclusion at this point is that demic diffusion predicts a speed compatible with the archaeological observations, whereas no cultural-diffusion model has been developed so far that can explain the observed speed.

### Interpolative Determination of the Most Likely Region of the Origin of Agriculture

**Figure 3:**
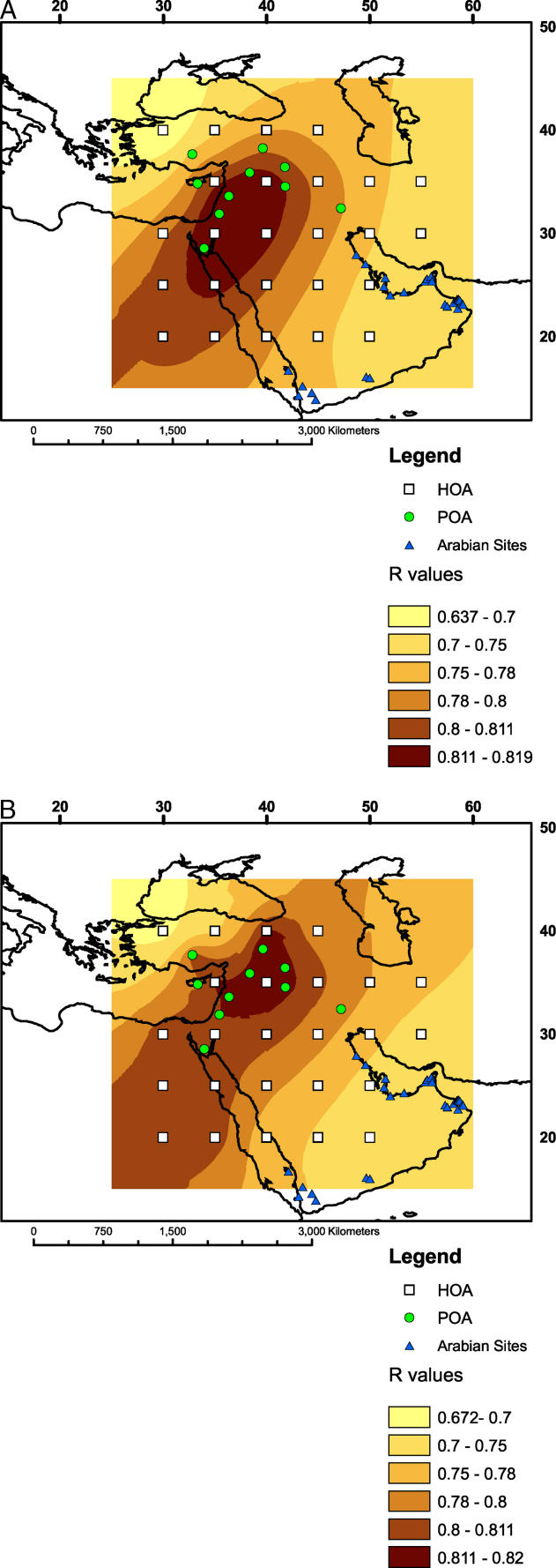
Interpolation Map of *R*-Values of HOAs (*n* = 765 Sites) Using great-circle distances (A) and shortest-path distances (B), these maps are based on uncalibrated dates and a slightly larger number of sites than those used in Figures 1 and 2 and Table 1. As a consistency test, the dataset now includes 30 Arabian sites. However, the results for the speed range are very similar to those obtained for the set of 735 sites. In addition, the use of calibrated dates does not lead to substantial changes in the maps (see the third and fourth figures in Protocol S1).

Finally, we consider a larger sample by adding 30 sites in Arabia (see Materials and Methods). The results of the HOA regressions are given in Table 2. The spatial distribution of these *R*-values was examined using ArcMap 8.3. We interpolated *R*-values using ordinary kriging (see Materials and Methods). We also checked that other methods of spatial interpolation (such as the Inverse Distance Method [32]) yield almost the same results. The two maps obtained by spatial interpolation of the *R*-values in Table 2 are presented in Figure 3A and 3B. They show varying grades or clines that differ in their steepness and geographic extent. The lighter a grade, the less likely it is that agriculture originated in that region. The darkest area is that with the highest interpolated value of *R* (*R* > 0.811; the lower limit, *R* = 0.811, was chosen in such a way that different zones can be clearly distinguished in Figure 3A and 3B). Here, progressively lighter grades surround the darkest area in an approximately concentric fashion. Using great-circle distances (Figure3A), the area of highest *R*-values focuses upon the Levant, and yet it also includes the north-west part of the Arabian Peninsula and the northern part of the Nile Valley. In terms of current archaeological knowledge, the latter are less likely to be involved in the origins of agriculture. Interestingly, this subregion disappears when shortest-path distances are used in the analysis (Figure 3B). When the two maps are compared, the most likely area is found to be located more to the north in the shortest-path analysis (Figure 3B). This is, in all likelihood, the better of the two maps for tracing the origins of agriculture. Figure 3B thus provides quantitative support for the view that agriculture is most likely to have originated in the area that today includes north-east Syria, northern Mesopotamia, and part of south-east Turkey near the site of Çayönü. The use of calibrated dates yields similar results (Protocol S1).

**Table 2:**
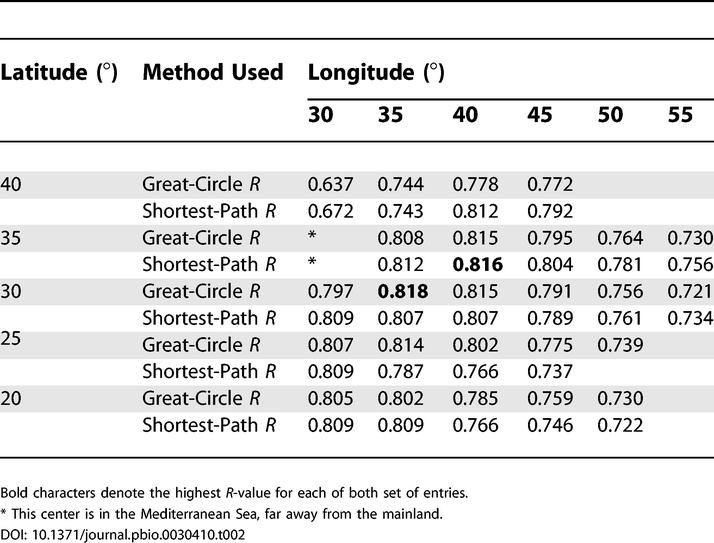
Correlation Coefficients, *R,* for HOAs and Uncalibrated Radiocarbon Dates (*n* = 765 Sites), Using Great-Circle Distances (Upper Entry in Each Cell, Used in Figure 3A) and Shortest-Path Distances (Lower Entry in Each Cell, Used in Figure 3B, See Text S1)

## Discussion

We estimated the overall speed of the spread to be in the range of 0.6–1.3 km/y. The *R*-values in Table 1 agree well with those reported by Ammerman and Cavalli-Sforza [5,7]. The correlation coefficients that they obtained (*R* = 0.89 for Jericho, *R* = 0.83 for Jarmo, *R* = 0.83 for Çayönü, *R* = 0.84 for Ali Kosh, and *R* = 0.86 for their center of gravity [our tenth POA]) are slightly higher than ours. This is not surprising since Ammerman and Cavalli-Sforza [5,7] chose to leave out sites in the Alps as well as those at high latitudes in northern Europe (to avoid the time delays in the arrival of early farming owing to the ecological adaptations called for in such places). They are included here, and this leads to an increase in the data dispersion (i.e., to lower *R*-values). This also explains why our speed range (0.7–1.1 km/y, using great circles and uncalibrated dates as did Ammerman and Cavalli-Sforza [5,7]), is slightly lower than theirs (the 53 sites and dates used by Ammerman and Cavalli-Sforza [5,7] yield 0.8–1.3 km/y, taking Jericho as the center [which yielded their highest *R*-value], with a 95% confidence interval). In agreement with Ammerman and Cavalli-Sforza [5,7], we find that a number of the correlation coefficients for our POAs are greater than 0.8 (see Tables 1 and 2). The implication here is that the phenomenon, as examined at the macro level (Europe as a whole), unfolded basically in a linear fashion (see the linear fits in Figure 2A and 2B). These results are particularly noteworthy, because the average rate for the spread (about 1 km/y) is now confirmed by a dataset that is some 15 times larger than the one used more than 30 y ago in the original analysis.

In addition, our rate of advance (0.6–1.3 km/y) is similar to the one determined by Gkiasta et al. [33], who obtained a speed of 1.3 km/y in their regression analysis. They did not estimate an error range for the rate—something that is essential if one intends to develop a comparative analysis of the observed speed and the speed predicted on the basis of a model (see Results). Their value for the correlation coefficient was *R* = 0.73—thus lower than ours. The differences between their rate (and their *R*-value) and ours may be due to the following: (1) all of their sites are more recent than 8,200 BP, whereas we included sites dating back to 11,000 BP; (2) they make the working assumption that the center of origin is Jericho, whereas we performed a more comprehensive analysis of the ten POAs shown in Figure 1 and then turned our attention to those with the highest *R*-values; and (3) we used 735 sites (a dataset about 50% larger than the one they used).

The observed rate (0.6–1.3 km/y, from Figure 2A and 2B) is consistent with that predicted by a *demic*-diffusion model (0.6–1.1 km/y, from equation 1). As mentioned earlier, we are not aware of any *cultural-*diffusion model that predicts a range consistent with the observed speed.

It is worth noting how slow the rate is on the ground (that is, in terms of a human generation). Although there is a tendency to imagine the spread racing across the map of Europe, it actually took more than 3,000 y (or 100 human generations) for the Neolithic transition to reach north-west Europe. What is involved—again on the macro level for Europe as a whole—is a slow, gradual process. At the same time, in the light of the early maritime spread of farming to Cyprus from the mainland, one can ask the following question: why did it then take almost 1,000 y to get to Crete, the next offshore island in the Mediterranean? At several sites on Cyprus, there is now good evidence for the arrival of the Neolithic package of domesticated crops and animals from the mainland by around 8,200 BC (calibrated). The fact that people were already using boats on a regular basis is also shown by the occurrence of obsidian (a volcanic glass used for making chipped-stone tools), which has its sources in Anatolia, at the same sites in Cyprus.

We reach much the same conclusion about the use of boats in the case of southern Italy, where obsidian from nearby islands is found at the oldest Neolithic sites in the region. Given the common use of boats in both parts of the Mediterranean, one might expect a faster rate for the spread between Cyprus and Italy than the one we observe. Why, in a maritime context, was the average speed in the central Mediterranean so slow? This is a puzzle that calls for further investigation. In fact, the slowness of the overall spread and its essentially linear character, as shown by the present analysis, may offer one of the best lines of argument for demic diffusion. Cultural diffusion can, and probably should, go faster. An excellent example is pottery, which appeared after the aceramic Neolithic and spread more rapidly than early farming [34].

The results of the great-circle analysis indicate that the area with the highest *R*-values (*R* > 0.811) encompasses the southern Levant and southern Mesopotamia; it contains five of the POAs (see Figure 3A). The shortest-path treatment, which takes into account the role of the Mediterranean as a barrier (see Text S2), gives an area with high *R*-values (*R* > 0.811), and this area shows a better match with the evidence regarding plant domestication since it does not include northern Egypt and the Red Sea. The area that it identifies as the most likely source for the spread of early agriculture is located in the northern Levant and the northern part of Mesopotamia; it contains six out of ten of the POAs (Figure 3B).

College et al. [25] examined the archaeobotanical remains recovered from 40 aceramic Neolithic sites in the Near East and south-east Europe. They note the similarity of most of the southern Levantine, Cypriot, and Aegean sites. They conclude that the contrast between these sites and those in Anatolia and the Euphrates Valley/central Steppe region of Syria points to the possibility of two dispersal routes. One route is a maritime-based colonization of Cyprus, central Anatolia, Crete, and Greece starting from a Levantine core region, as previously suggested by Van Andel and Runnels [35]. The second route is a land route from central/western Anatolia, reaching Thrace and south-east Europe. It would be of interest, in future work, to try to test statistically the two-route model put forward by College et al. [25].

Pinhasi and Pluciennik [26], in their analysis of craniometric affinities between populations, point to the homogeneity between Çatal Höyük and early Neolithic Greek and south-eastern European groups. This homogeneity contrasts with the pronounced heterogeneity found among other Pre-Pottery Neolithic groups in the Near East. On the basis of these results, they hypothesize that a founder population from central Anatolia (represented by specimens from Çatal Höyük) spread into south-east and central Europe. The results of the shortest-path analysis of the POAs could be consistent with their position, since they suggest that Çatal Höyük falls in the region adjacent to the one with the maximum *R*-values (Figure 3B).

We concur with Özdog˘an's assertion that “an unbiased reassessment of the evidence strongly implies that there were multiple paths in the westward movement of the Neolithic way of life” ([36], pp. 51–52). Aceramic Neolithic levels at sites on Cyprus (late ninth millennium BC [calibrated]), Crete and the Argolid (eight and early seventh millennia BC [calibrated]) are strongly suggestive of an initial population dispersal wave from one or more centers in the Near East [37]. At the present time, it is unclear whether farming reached south-east Europe by means of a secondary demic expansion from Anatolia or as a continuation of the initial dispersal involving Cyprus, Crete, and mainland south-east Greece. In any event, Figure 3B does provide, at this stage of research, spatial information regarding differing grades of likelihood for tracing the origins of agriculture.

In closing, we would like to stress again that our aim here is not to deny the existence of regional variability in Europe, nor to deny that local populations of late hunters and gatherers may have made a significant contribution to the Neolithic transition in certain regions [38,39]. On the other hand, for many areas of southern Europe, it remains an open question as to whether or not local populations of foragers were actually living there in the time just before the Neolithic transition. In countries such as Greece and Italy, where many archaeological surveys have been carried out over the past 30 y, very few late Mesolithic sites have come to light so far [40]. Our purpose here is to return to the big picture. Indeed, the pattern of dispersion shown in Figure 2B implies that the processes involved may have been extremely complex and at least to some extent geographically non-homogeneous. This is precisely why it is important to consider more fully what makes it possible for the very simple formula shown in equation 1 to account for the average rate of spread over Europe. While our analysis takes a mathematical approach to the overall Neolithic spread, and by doing so, we are not in a position to tackle the question of a mosaic of regional processes, we nevertheless think that the high *R*-values obtained in our new analysis show that, at the macro level of human population biology, the wave-of-advance model is not just a mathematical artifact. Rather, it points to an overall spatiotemporal pattern in the spread of the Neolithic lifestyle, which best agrees with an initial dispersal from the Levantine/Mesopotanian core region to Europe, and which does not exclude subsequent range expansions, colonizations, and jump dispersals.

## Materials and Methods

### Samples

Our radiocarbon dataset includes uncalibrated dates of the earliest Neolithic occupation from the earliest-dated levels of 735 sites in the Near East, Europe, and Asia (see Figure 1), as well as from 30 Arabian sites (all dates are available, see Table S1). The dates were obtained from the following online databases: UK Archaeology Data Service (http://www.ads.ahds.ac.uk, Canew database (http://canew.org, Near Eastern Radiocarbon Context database (http://www.context-database.de, and the Radon Workgroup database (http://www.jungsteinsite.de/radon/radon.htm). We selected the earliest date of Neolithic occupation for each site. We used only dates that have standard errors of the mean of less than 200 radiocarbon years. We omitted all dates with higher error intervals, as well as outlier dates (i.e., early occupation dates that are rejected by most archaeologists as being erroneously too early or too late, see Table S2). We preferred, whenever possible, to take dates coming from charcoal or bone collagen rather than shells. The sites and corresponding dates provide a secure sample for the earliest appearance of each of the early Neolithic archaeological cultures of Europe (such as the Linienbandkermik, Starçevo, Körös, Çris, Cardial, and so forth). Dates from the Alpine and Scandinavian regions were also included, despite the late appearance of Neolithic occupation in these regions owing to the “delayed” adoption of agriculture in these zones for environmental reasons [5].

Our basic approach to the analysis is to include all of the robust data that are available, without trying to be more selective or rigorous about dates that give a good estimate of the first appearance of the Neolithic in a given area. On the one hand, there is a virtue in this approach: one avoids bias (making choices that are arbitrary). On the negative side, however, one includes some data that are weak. The measured correlation coefficients will be lower, in all likelihood, than those obtained by using only high-quality data (for example, accelerator mass spectrometry [AMS] dates). AMS dates have two main advantages: (1) they can be obtained directly from seeds and bones, and (2) the smaller size means that one is often in a position to date samples of higher quality than previously. There is a consensus that AMS dating represents a major advance for the study of the Neolithic transition in Europe [40]. Out of our total of 765 dates, comparatively few have been obtained by AMS. Clearly, a relevant point is the trade-off between the quality and the quantity of the dates used. In measuring the overall rate, this probably makes little difference. For the estimation of regional rates in Europe (not the aim of the present paper), it may make more of a difference. The advantage of our global approach is the sheer quantity of data that are available today.

In Table 1, Center 1 has been investigated by Pinhasi [41]. Center 4 (Jericho) has been considered, among others, by Gkiasta et al. [33]. We chose the other centers (Centers 1–9) on the basis of early Neolithic sites that are POAs [42]. For comparative purposes, we also include Center 10, which was proposed by Ammerman and Cavalli-Sforza [5,7]: Center 10 is the geographic center of gravity of the sites of Jericho, Jarmo, Cayönü, and Ali Kosh.

In Table 2 and Figure 3, we added 30 sites in Arabia to the 735 sites used in Table 1 and Figure 2, primarily as a consistency test of the results from Figure 2A and 2B. We chose not to include sites in North Africa because they are much more controversial and are few in number [43,44]; some of them also have problems associated with their chronometric provenance. In addition, there are no reliable dates from Egypt and the Sahara, as the Neolithic occupation there involved nomadic tribes with domesticated cattle (and in some cases pottery, but without permanent dwellings and other Neolithic criteria such as early cereals). In any case, even if reliable dates become available in the future, using North-African sites will presumably not significantly change the interpolative *R*-value maps (see Figure 3A and 3B) as they are located far to the west of the Near Eastern region. In contrast, the interpolated maps are sensitive to the inclusion of the 30 Arabian sites because they are geographically close to the Near East (moreover, there are no other early sites further to the south). In any case, when the 30 Arabian sites were included, we obtained the same speed range (0.6–1.3 km/y) as that obtained above with the 735-site sample only. Therefore, we can say that the 30 Arabian sites serve as a self-consistency check of the speed range computed.

### Calibration

Uncalibrated radiocarbon dates are based on the premise that the atmospheric ratio of carbon-14 to carbon-12 has been constant over time. However, this premise is only approximately valid. Briefly, carbon-14 dates can be calibrated by using tree-ring, glacial, ice-core, and other known climatic sequences. We applied the CalPal calibration software package (www.calpal.de) to all dates and their standard errors of the mean, using specifically the CalPal January 2004 calibration curve, which is based on six datasets comprising tree-ring, lacustrine, and glacial data. For more details, together with figures corresponding to Figure 2A and 2B and Figure 3A and 3B, but obtained using calibrated instead of uncalibrated dates, see Protocol S1. It can be seen in Protocol S1 that the figures do not change appreciably after calibration is taken into account, so that the conclusions remain the same.

### Distances computations

A great-circle distance between two geographic points is the shortest distance along the circle on the Earth's surface (considered as a sphere) that contains both points. Shortest-path distances take into account the fact that some great-circle distances are not realistic, owing primarily to the presence of the Mediterranean Sea in our case (Text S2).

### Statistical analysis

We calculated two linear regressions for each of the ten POAs in Figure 1, namely the distance-against-date and the date-against-distance regressions. Both were computed using the radiocarbon dates of the 735 sites and their distances (in km) from a given POA. The distance-versus-date regression corresponds to fitting a linear model to predict the position (distance) of the front of the population spread after a given time has elapsed, but it also makes sense to fit a linear model the other way (date-versus-distance). It corresponds to predicting the time that it takes for the population wave front to travel a given distance. Combining both fits is useful in order to estimate a wider, more reasonable, error range for the observed speed (see Figure 2A, caption). Comparing the speed predicted by a theoretical model (in this case, a demic reaction-diffusion model, by means of equation 1) to the observed speed is much more meaningful if, as is done here, the error ranges of both the observed and predicted speeds are determined.

### Spatiotemporal analysis

The interpolative method of ordinary kriging [32] (used in Figure 3A and 3B) takes into account the *R*-values at surrounding locations in order to obtain the *R*-value at another location. This method fits the *R*-values to a sum of two functions. The first function is a polynomial of latitude and longitude. The second function has zero average, and its difference between two spatial points does not depend on their locations but only on the distance between them. This second function is used in an attempt to control for autocorrelation between the values of a geographic variable at nearby points, a basic principle in geography [32]. We also found that other methods of spatial interpolation yield almost the same result as that shown in Figure 3A and 3B (e.g., the Inverse Distance Method, where the second function does not make use of autocorrelation but instead makes use of a simple algorithm based on distance [32]).

## Supporting Information

Protocol S1 Computation and Effect of Calibrated Dates Protocol S1 includes four figures, which correspond with and are very similar to, Figure 2A and 2B and Figure 3A and 3B, but use calibrated (instead of uncalibrated) dates. Therefore, using calibrated dates does not lead to any substantial change in the results and conclusions made here.
(474 KB PDF).

Table S1 Information about 765 Neolithic Sites Latitude/longitude, radiocarbon date, and additional archaeological information.
(1.1 MB XLS).

Table S2 List of Discarded Sites See Materials and Methods.
(15 KB XLS).

Text S1 Neolithic Data An explanation of the entries included in Table S1 is presented.
(102 KB PDF).

Text S2 Computation of Shortest-Path Distances An explanation is presented of the approach that we used to compute the shortest-path and great-circle distances included in Table S1. Comparisons are provided of a great-circle distance, the corresponding shortest-path-on-Earth distance, and the intermediate approach we used. The latter computes shortest-path distances taking into account the possibility of some sea travel (as implied by the presence of early Neolithic sites on islands such as Cyprus, Crete, Lipari, and Sardinia) but not long-distance voyaging (e.g., from one end of the Mediterranean to the other).
(165 KB PDF).

Text S3 Demographic Data Comparison and comment are presented with regard to the demographic data available for the determination of the parameters *a*, *m,* and *T,* which are used in equation 1 to predict the speed of the wave of advance.
(99 KB PDF).

## Abbreviations

AMS: accelerator mass spectrometry
BP: before present
HOA: hypothetical center of origin of agriculture
POA: probable center of origin of agriculture
