# Supplementary material for: Tracing the Origin and Spread of Agriculture in Europe
Source: PLoS Biol. 2005 Nov 29;3(12):e410. doi: 10.1371/journal.pbio.0030410 (PMC1287502; doi:10.1371/journal.pbio.0030410)

Supporting Protocol S1: Computation and effect of calibrated dates

Uncalibrated radiocarbon dates are measured in conventional radiocarbon years that are based on the premise that the atmospheric ratio of carbon-14/carbon-12 ratio has been constant (in our case, during the last 11,000 years). As there is appreciable divergence between the radiocarbon years and calendar years as one goes back in time beyond 500 BC, there is a need to calibrate the former using tree-ring, glacial, ice-core and other known climatic sequences. Calibration programs allow one to calibrate dates with corresponding standard errors applying a combination of data sets and to examine those against various calibration curves. We applied CalPal calibration software package (www.calpal.de) to all dates and their standard errors using the CalPal 2004_Jan calibration curve which is based on six tree-ring, lacustrine, and glacial data sets.

The following figures (Figs. 2c, 2d, 3c and 3d) are the same as Figs 2a, 2b, 3a and 3b in the paper, but using calibrated instead of uncalibrated dates. It is seen that they are very similar to the corresponding figures in the paper. Thus, the conclusions of our work remain valid if calibrated dates are used.

Fig. 2c

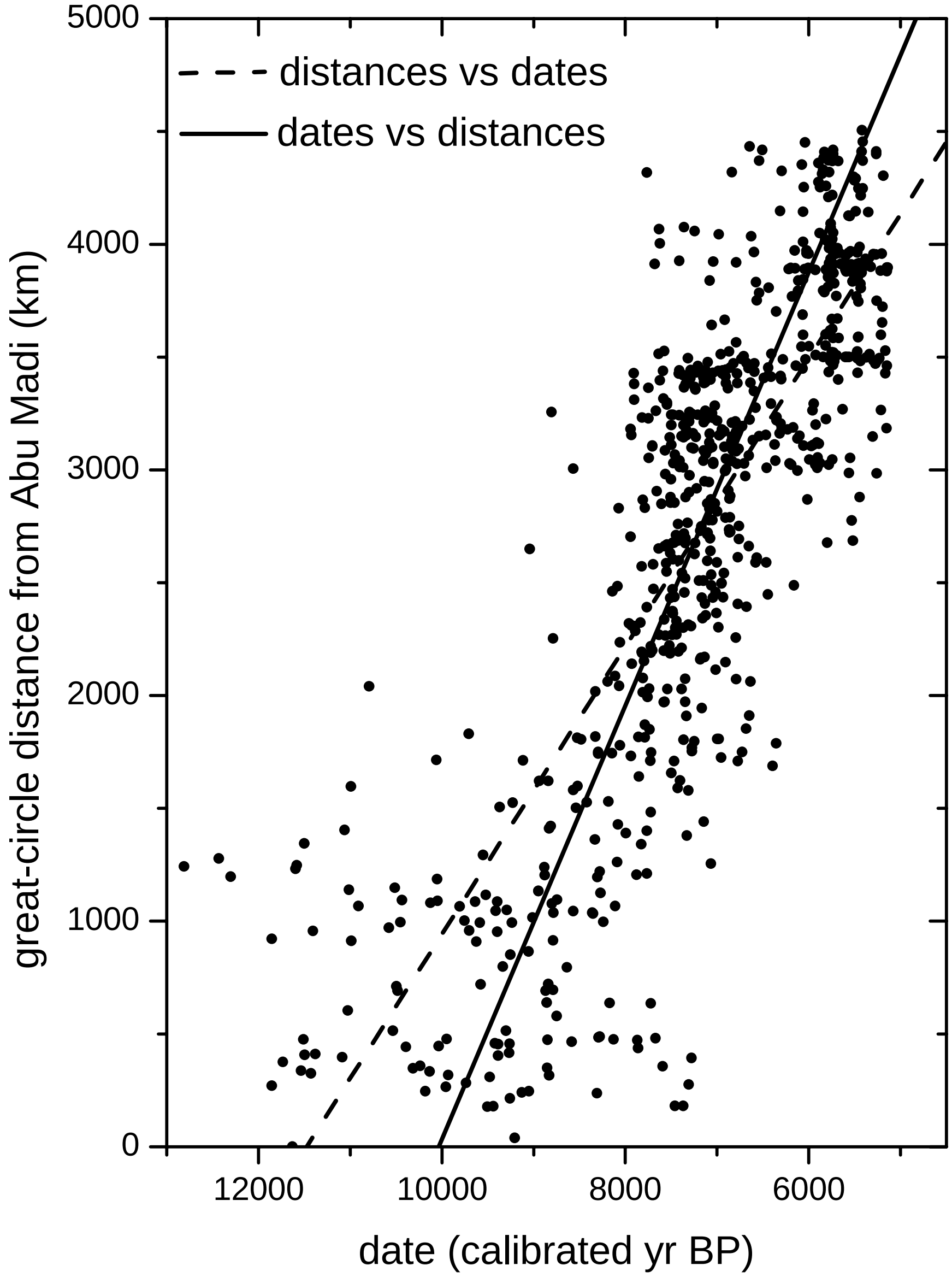

Figure 2c. Linear regression fits to the data for calibrated dates and great-circle distances of sites computed from the POA centre with the highest value of R of those in Table 1 (Abu Madi). The distances-vs-dates regression yields (0.64±0.03) km/yr, whereas the dates-vs-distances regression yields (0.96±0.05) km/yr. The overall estimated speed range it thus 0.6-1.0 km/yr. This range is very similar to that determined using uncalibrated dates (namely 0.7-1.1 km/yr, from Fig. 2a in the paper).

Fig. 2d

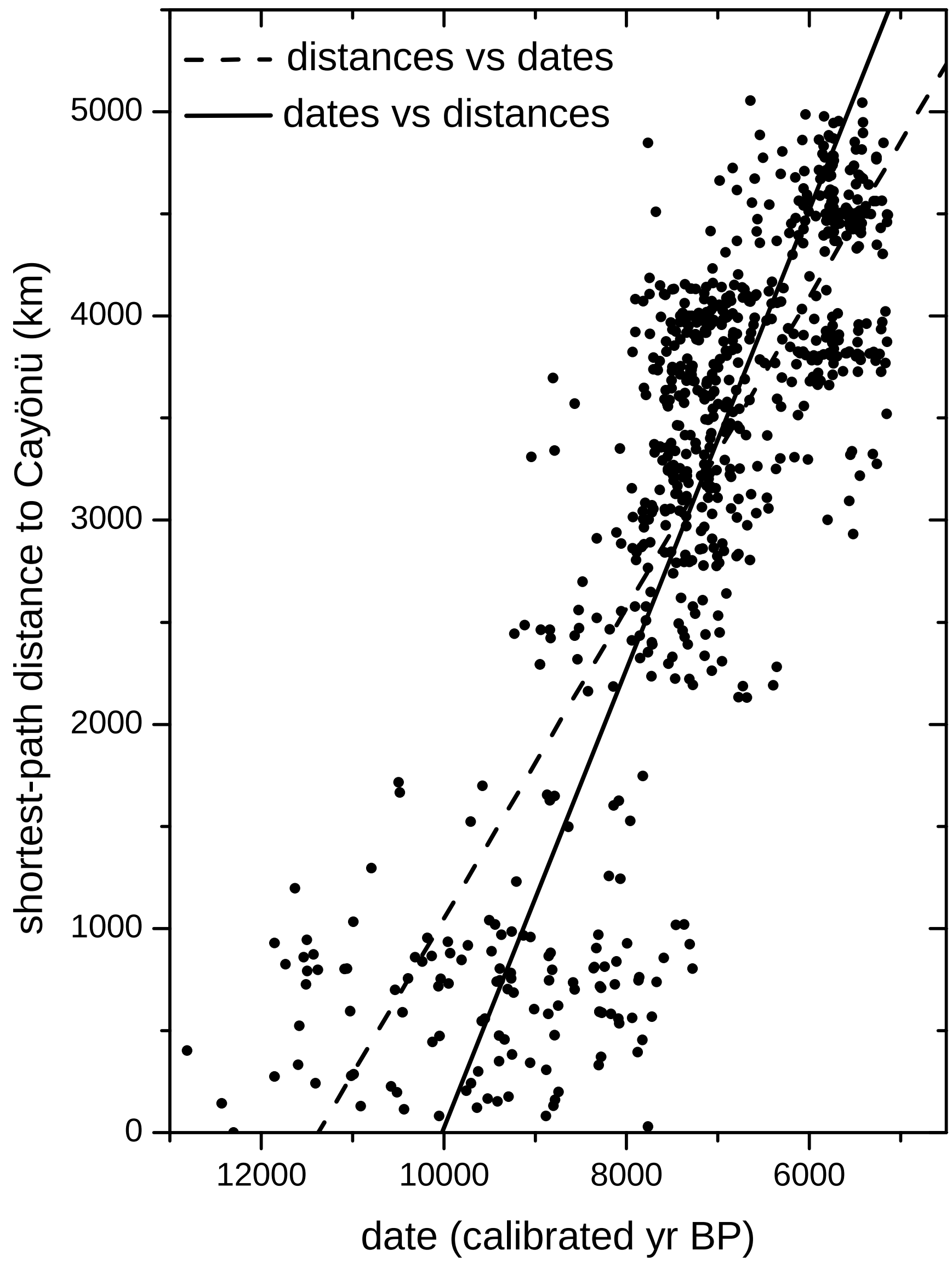

Figure 2d. Linear regression fits to the data for calibrated dates and shortest-path distances of sites computed from the POA centre with the highest value of R of those in Table 1 (Cayönü). The distances-vs-dates regression yields (0.76±0.04) km/yr, whereas the dates-vs-distances regression yields (1.12±0.06) km/yr. The overall estimated speed range it thus 0.7-1.1 km/yr. This range is very similar to that determined using uncalibrated dates (namely 0.8-1.3 km/yr, from Fig. 2b in the paper).

Figure 3c. Interpolative map of R values, using great-circle distances and calibrated dates. This figure is the same as Fig. 3a in the paper, but using calibrated instead of uncalibrated dates. Note that the region of highest values of R implied by this figure is very similar to that in Fig. 3a in the paper.

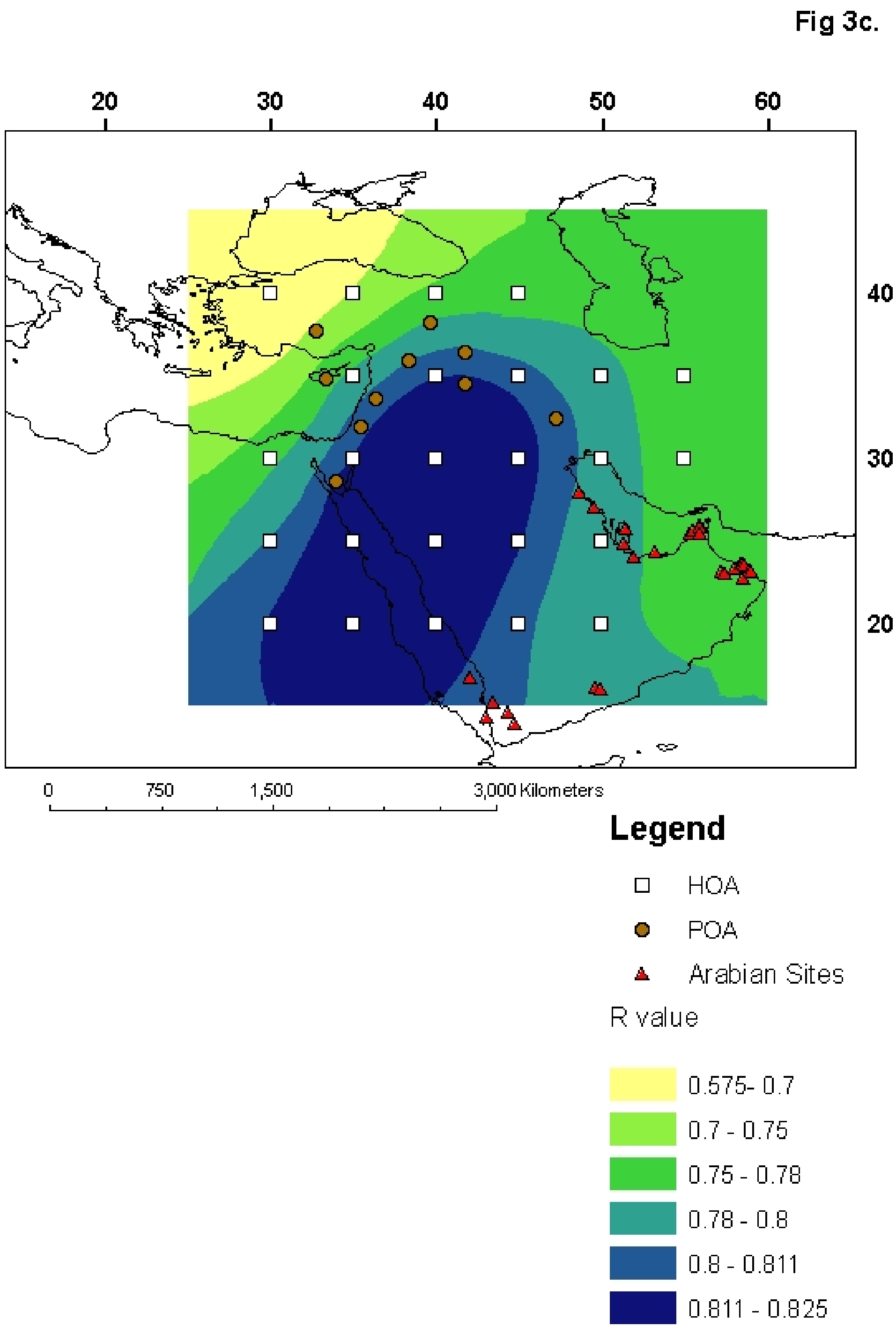

Figure 3d. Interpolative map of R values, using shortest-path distances and calibrated dates. This figure is the same as Fig. 3b in the paper, but using calibrated instead of uncalibrated dates. Note that the region of highest values of R implied by this figure is very similar to that in Fig. 3b in the paper.

Fig 3d.

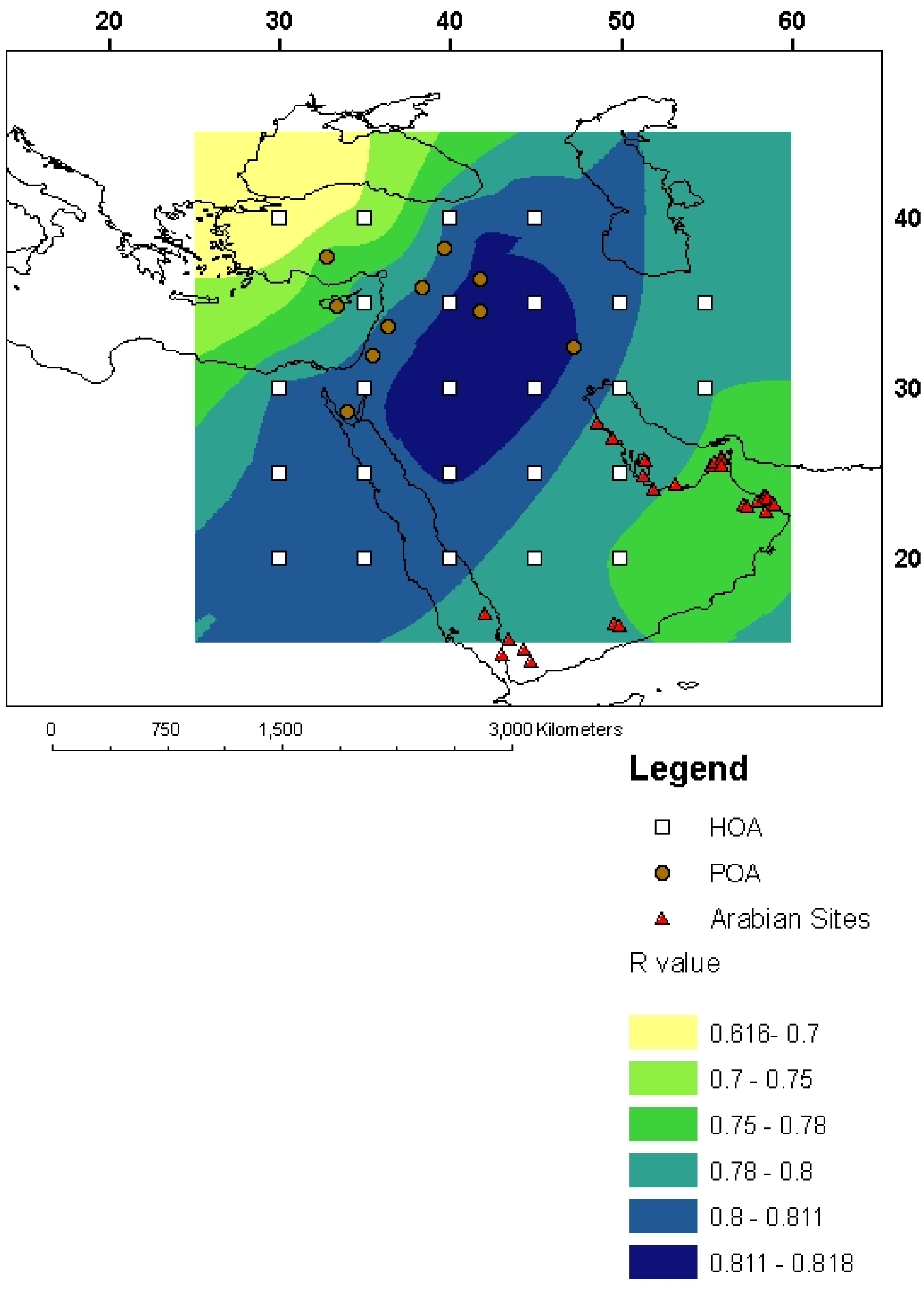

Supplement: Supplementary file 1 [file pbio.0030410.sd001.pdf]
