# Supplementary material for: Tracing the Origin and Spread of Agriculture in Europe
Source: PLoS Biol. 2005 Nov 29;3(12):e410. doi: 10.1371/journal.pbio.0030410 (PMC1287502; doi:10.1371/journal.pbio.0030410)
Supplement: Supplementary file 2 [file pbio.0030410.sd002.pdf]

Supporting Text S1: Neolithic data

Table S1 contains information for 765 Neolithic sites: Lat/Long, radiocarbon date & additional archaeological information (rows 40 to 804).

In Table S1 the locations of all 35 centres used (25 HOAs and 10 POAs) are given (rows 5 to 39).

Also given in Table S1 are the locations of two additional points used: point A/S (on the Anatolian/Syrian border), and point S/F (on the Spanish/French border) (rows 3 and 4).

The main entries in Table S1 are:

· Column J ("Uncal C14 BP") gives the earliest uncalibrated radiocarbon date for Early Neolithic context.

· Column L ("CAL C14 BP") is the calibrated date using Oxcal 2004 software (www.oxcal.de).

· Columns Q ("HOA1") to AY ("Ali Kosh") give **shortest-path** distances (in km) to the corresponding centre.

· Columns AZ ("GHOA1") to CH ("GAli Kosh") give **great-circle** distances (in km) to the corresponding centre.

Shortest-path distances (columns Q to AY) have been computed as explained in Supporting Text S2, specially Fig S1:

· Sites in Arabia, Near East, Anatolia and Asia (as stated in column E): great-circle distance to each centre.

· European non-Iberian sites ("Europe" in column E): great-circle distance from the site to the A/S point, plus great-circle distance from this point to each centre (see e.g. the green line in Fig S1).

· Iberian sites ("Iberia" in column E): great-circle distance from the site to the S/F point, plus great-circle from this point to the A/S point, plus great-circle from this point to each centre (see e.g. the red line in Fig S1).

Notes:

1. Columns S, T, AM, AN & AO (Egyptian centres: HOA3, HOA4, HOA23, HOA24 & HOA25): distances have been computed as explained above, but relative to a point on the Egypt/Israel border (Lat=31º, Long=34.5º), and the great-circle distance from this point to the HOA has been added.

2. Columns R, U, AA, AF; AP & AV (HOA 2, HOA 5, HOA11, HOA16; POAs Çatal Höyük and Cayönü in Anatolia): distances have been computed as explained above, but relative to the Dardanelles (Lat=41º, Long=29º), and the great-circle distance from the Dardanelles to the centre has been added.

The following table is included as an attempt to further clarify the shortest-distance computation approach used. It is exactly the same as Table 2 in the paper, but using colour codes for the criteria used to compute short distances (according to the location of HOAs).

Table 2. Correlation coefficients R for hypothetical origin of agriculture (HOA) centres (see Fig. 3) using uncalibrated radiocarbon dates (N = 757 sites) using great-circle (top) and shortest-path distances (bottom). Bold characters are used for the two highest R values for each method.

**great-circle R**<br><br>**shortest-path R**	Long = 30º	Long = 35º	Long = 40º	Long = 45º	Long = 50º	Long = 55º	Long = 60º
Lat = 40º	0.637<br>0.672	0.744<br>0.743	0.778<br>0.812	0.772<br>0.792			
Lat = 35º	*<br>*	0.808<br>0.812	0.815<br>**0.816**	0.795<br>0.804	0.764<br>0.781	0.730<br>0.756	
Lat = 30º	0.797<br>0.809	**0.818**<br>0.807	0.815<br>0.807	0.791<br>0.789	0.756<br>0.761	0.721<br>0.734	
Lat= 25º	0.807<br>0.809	0.814<br>0.809	0.802<br>0.787	0.775<br>0.766	0.739<br>0.737		
Lat= 20º	0.805<br>0.809	0.802<br>0.809	0.785<br>0.766	0.759<br>0.746	0.730<br>0.722		

* This centre is in the Mediterranean Sea, far away from the mainland.

Shortest-path distances (lower entries) have been computed as follows:

Red:
· Non-Iberian European sites: via the Anatolia/Syria point (Lat=36.75º, Long=36.1º) [see Supp. Text S2, green line in Fig S1].
· Anatolian & Near Eastern sites: shortest path equal to great circle.

Blue:
· Non-Iberian sites west of the Dardanelles: via the Dardanelles point (Lat=41º, Long=29º).
· Anatolian & Near Eastern sites: shortest path equal to great circle.

Green:
· Anatolian & Near Eastern sites: via the Sinai point (Lat=31º, Long=34.5º).
· Non-Iberian European sites: via the Anatolia/Syria point and the Sinai point.

Note: In all cases, for Iberian sites shortest paths go through the Spain/France point (Lat=42.4, Long=3.25º) [see Supp. Text S2, red line in Fig S1].
