# Supplementary material for: Tracing the Origin and Spread of Agriculture in Europe
Source: PLoS Biol. 2005 Nov 29;3(12):e410. doi: 10.1371/journal.pbio.0030410 (PMC1287502; doi:10.1371/journal.pbio.0030410)
Supplement: Supplementary file 3 [file pbio.0030410.sd003.pdf]

Supporting Text S2: Computation of shortest-path distances

1. A great-circle route is defined as the shortest path between two geographic points on the Earth, considered a sphere [1]. Therefore, the great-circle route does not take into account the presence of geographic barriers such as seas, mountains, etc. (for example, the blue line in Fig. S1 is the great circle path between Jericho and an Italian site).

2. In our case, the Mediterranean sea is clearly the main geographic barrier. At first sight, it might seem that the right way to take it into account is to compute shortest-path distances on the land (yellow line in Fig. S1). But this approach cannot be applied to the sites on islands (such as Cyprus, Crete, Sicily, Corsica, etc.) in Fig. S1. Also, the presence of Neolithic sites on islands makes it inconsistent to neglect any possibility of sea navigation. Therefore, the length of the yellow line in Fig. S1 is unrealistically high.

3. For this reason, we followed an intermediate approach between those in paragraphs 1. and 2. above. Our approach leaves out very long sea travels (such as that implied by the blue line in Fig. S1) but allows for short sea travels, and is therefore consistent with the presence of Neolithic sites on some islands (green line in Fig. S1). The results can be found in Table S1, columns Q to AY (see Supporting Text S1 for a detailed explanation of that table). We have also tried some additional intermediate approaches (e.g., allowing for a sea travel between Jericho and a point in western Anatolia in Fig. S1), and checked that they lead only to small changes in our final results.

4. The pattern of obsidian exchange in the case of the earliest Neolithic sites on Cyprus and in the case of the early Neolithic sites in peninsular Italy (all four of the obsidian sources used at the sites occur on islands -- Sardinia, Palmarola, Lipari and Pantellaria) indicate a regular/frequent practice of navigation. In short, the use of boats by coastal people is not a rare or unusual event in the Mediterranean. On the other hand, people did not set out on long distance voyages from one end of the Mediterranean to the other. These are additional motivations for our approach (point 3. above) to the computation of shortest-path distances.

[1] We computed great-circle distances using ArcView 3.2 with the extension 'Azimuth and Distance Matrix, v. 1.4' [2]. This extension calculates a distance matrix from a set of points (e.g., sites and centers). The great-circle distance between two points, determined in this way, is the shortest distance along the circle on the Earth surface (considered a sphere) which contains both points, as in Ammerman & Cavalli-Sforza (1971) original work. The results can be found in Table S1, columns AZ to CH (see Supporting Text S1).

[2] Jenness, J. 2004. Distance Matrix (dist_mat_jen.avx) extension for ArcView 3.x, v. 1.4. Jenness Enterprises. Available at: http://www.jennessent.com/arcview/dist_matrix.htm via the internet. Accessed 2004 Dec 15th.

Fig S1

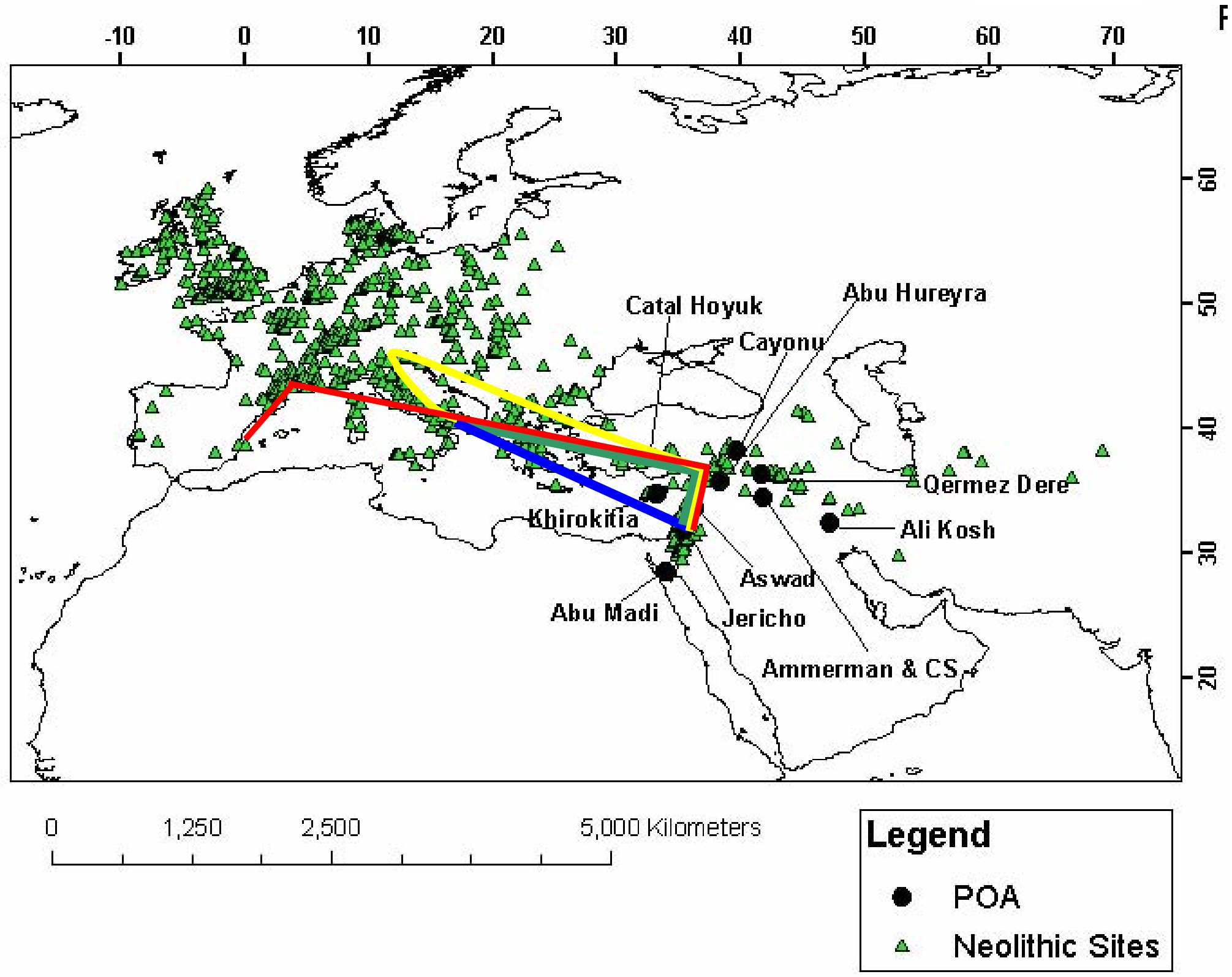

Fig. S1 Comparison between great-circle distance (blue), shortest-path land distance (yellow) and the intermediate path we have used to compute shortest-path distances (green). In this example the origin is Jericho and the final point is an Italian site. Our intermediate shortest-distance path for an Spanish site is also shown (red). We have computed shortest-path distances for all other cases analogously to the green and red paths on the map (see Supporting Text S1).
