# Supplementary material for: Tracing the Origin and Spread of Agriculture in Europe
Source: PLoS Biol. 2005 Nov 29;3(12):e410. doi: 10.1371/journal.pbio.0030410 (PMC1287502; doi:10.1371/journal.pbio.0030410)
Supplement: Supplementary file 4 [file pbio.0030410.sd004.pdf]

Supporting Text S3: Demographic data

The speed of the wave of advance is given by Eq. (1) in our paper. In that equation, the parameters *a*, *m* and *T* appear. Below we gather all sources in the literature known to us such that data useful to compute these parameters appear. Very few populations have been found for which such data are available, so most estimations published in the literature have been performed with an 80% confidence-level interval.

1. Parameter *a* (initial growth rate of the population number).

This parameter can be estimated if the numbers of populations who settled in empty space are known as a function of time. Such data were reported by Birdsell [1] for two different populations: Pitcairn Island (located about 4,000 miles west of Chile, dates 1790-1856) and the Bass Strait islands (between Australia and Tasmania, dates 1820-1945). Birdsell plotted the population numbers against the elapsed time. He found that the points of both datasets fall on almost exactly the same curve. This is impressive because they correspond to two populations widely separated in space and time. By fitting an exponential increase in population number P (i.e., $P = P_0 e^{at}$) to those data, the 80% confidence-level range for *a* has been estimated [2] as 0.029-0.035/yr. This is the range used in our paper.

An additonal interesting source can be found in Lotka's seminal book [3], where he fits a logistic to the population numbers of the United States (dates 1790-1910). In this way he obtained a value for $a = 0.0313$/yr, which is completely consistent with the range above. Note, however, that it includes immigration as well as internal growth of the population number. Other examples mentioned in the literature are the European settlers in French Canada and South Africa [4], but we are not so far aware of detailed data for their population numbers versus time, so that we have not been able to fit a curve and estimate with statistical confidence their values of *a*.

2. Parameter *m* (mobility of the population)

This parameter is equal to the mean squared displacement per generation [2]. Here the distance squared and averaged is that between the birthplace of a person and the birthplace of his parent or mother (not the distance between the birthplaces of the father and that of the mother). The data necessary to estimate *m* are given in the form of an histogram of the numbers (or percentages) of people versus distance moved. Such data are known for present populations. But primitive ones may be reasonably expected to have rather different mobility patterns. Moreover, the few hunter-gatherers which still exist also seem to have quite a different mobility than primitive farmers [5]. All data so far known to us for primitive farmers are those of three Majangir populations, a shifting agriculturalist people in southwestern Ethiopia [6]. They convert forest into fields, cultivate them for e.g. four years (until they loose productivity), and then leave the forest grow again for e.g. ten years (they do not use manure). Their mean mobilities have been estimated [7]. The corresponding 80% confidence-level range is 900-2200 km^2/gen. This is the range used in our paper. By mobility one here refers not to the annual slight shifting (as required by their system of agriculture), but to intergenerational (mainly discontinuous) changes of residence [6]. The soil used by the Majangir is very fertile, with abundant precipitation and excellent drainage. Similarly, the first Neolithics tended to choose very good land, which had never been cultivated before. Therefore, it seems reasonable to expect that the mobility of the first Neolithics was more similar to that of the Majangir than to that of historical European populations.

3. Parameter *T* (generation time)

A theory with distributed delays has recently been proposed [8]. It makes it absolutely clear that *T* is the *mean* age difference between a person and *all* of their children, not just with the *oldest* one (the latter difference was used for *T* in the first papers on the time-delayed theory Neolithic transition [2], and is sometimes called the generation time in demography). A statistical analysis of the values of *T*

for the Majangir [6] yields the range 29-35 yr (80% confidence-level interval). This is the range used in our paper.

References

[1] Birdsell JP (1957) Some population problems involving Pleistocene man. Cold Spring Harbor Symp. Quant. Biol. 22: 47-69, specially Fig. 1.

[2] Fort J, Méndez V (1999) Time-delayed theory of the Neolithic transition in Europe. Phys Rev Lett 82: 867-871.

[3] Lotka aJ (1956) *Elements of Mathematical Biology* (New York: Dover)

[4] Cavalli-Sforza LL (2002) Demic diffusion as the basic process of human expansions. In *Examining the farming/language dispersal hypothesis*. Edited by P. Bellwood and C. Renfrew, pp. 79-88. Cambridge: McDonald Institute for Archaeological Research.

[5] Fort J, Pujol T, Cavalli-Sforza LL (2004) Palaeolithic populations waves of advance. Camb. Archaeol. J. 14: 53-61, specially table 1.

[6] Stauder (1971) *The Majangir: Ecology and society of a southwest Ethiopian people* (Cambridge University Press).

[7] Ammerman AJ, Cavalli-Sforza LL (1984) The Neolithic transition and the genetics of populations in Europe. Princeton, NJ: Princeton University Press, specially p. 155.

[8] Fort J, Jana D, Humet J. (2004) Multi-delayed random walks. Theory and application to the Neolithic transition in Europe. Phys Rev E70: 031913, 1-6.
